# 阿美替尼联合贝伐珠单抗治疗晚期NSCLC伴原发EGFR T790M突变：3例病案报道及文献复习

**DOI:** 10.3779/j.issn.1009-3419.2023.101.04

**Published:** 2023-02-20

**Authors:** Xue YANG, Fanlu MENG, Diansheng ZHONG

**Affiliations:** 300052 天津，天津医科大学总医院肿瘤内科; Department of Medical Oncology, Tianjin Medical University General Hospital, Tianjin 300052, China

**Keywords:** 阿美替尼, 原发T790M突变, 贝伐珠单抗, 肺肿瘤, Aumolertinib, Primary T790M, Bevacizumab, Lung neoplasms

## Abstract

随着检测技术的发展，非小细胞肺癌患者（non-small cell lung cancer, NSCLC）伴原发表皮生长因子受体（epidermal growth factor receptor, EGFR）T790M突变的检出率不断增加，而针对原发EGFR T790M突变NSCLC的一线治疗尚无标准。本文中我们报道了3例晚期NSCLC伴EGFR敏感突变及原发T790M突变的治疗经验，3例患者一线初治均为阿美替尼联合贝伐珠单抗。其中，1例在治疗3个月后因出血风险停用贝伐珠单抗并于10个月后更换为奥希替尼；1例患者在治疗13个月后更换为奥希替尼并停用贝伐珠单抗。3例患者最佳疗效均达部分缓解（partial response, PR）。随访至2022年10月，2例患者一线治疗后进展，无进展生存期（progression-free survival, PFS）分别为11个月和7个月；1例患者治疗后持续反应，治疗时间已达19个月。2例患者基线伴有多发脑转移，一线治疗后颅内病灶最佳疗效均为PR，颅内PFS分别为14个月和未达到（16^+^个月）。3例患者在治疗期间未见新的不良反应，未发生3级以上不良反应。此外，我们总结了NSCLC伴原发EGFR T790M突变的研究进展。总之，阿美替尼联合贝伐珠单抗初始治疗晚期NSCLC伴原发EGFR T790M突变具有较高的客观缓解率（objective response rate, ORR）及颅内病灶的控制能力，可作为晚期NSCLC伴原发EGFR T790M突变的一线治疗。

## Abstract (en)

With the development of sequencing technology, the detection rate of non-small cell lung cancer (NSCLC) with primary epidermal growth factor receptor (EGFR) T790M mutation is increasing. However, the first-line treatment for primary EGFR T790M-mutated NSCLC still lacks standard recommendations. Here, we reported three advanced NSCLC cases with EGFR-activating mutation and primary T790M mutation. The patients were initially treated with Aumolertinib combination with Bevacizumab; among which, one case was discontinued Bevacizumab due to bleeding risk after treatment for three months. Treatment was switched to Osimertinib after ten months of treatment. Another case switched to Osimertinib and discontinued Bevacizumab after thirteen months of treatment. The best effect response in all three cases was partial response (PR) after initial treatment. Two cases progressed after first-line treatment and progression-free survival (PFS) was eleven months and seven months respectively. The other one patient had persistent response after treatment, and the treatment duration has reached nineteen months. Two cases had multiple brain metastases before administration and the best response to intracranial lesions was PR. The intracranial PFS was fourteen months and not reached (16^+^ months), respectively. There were no new adverse events (AEs), and no AEs of grade three or above were reported. In addition, we summarized the research progress of Osimertinib in the treatment of NSCLC with primary EGFR T790M mutation. In conclusion, Aumolertinib combined with Bevacizumab in the treatment of advanced NSCLC with primary EGFR T790M mutation has a high objective response rate (ORR) and control ability of intracranial lesions, which can be used as one of the initial options for first-line advanced NSCLC with primary EGFR T790M mutation.

表皮生长因子受体（epidermal growth factor receptor, EGFR）获得性T790M突变为一代、二代EGFR酪氨酸激酶抑制剂（tyrosine kinase inhibitors, TKIs）耐药的主要机制，其发生率为50%-60% ^[1,2]^。而原发EGFR T790M的突变率因检测方法敏感度不一导致波动较大。在非小细胞肺癌（non-small cell lung cancer, NSCLC）中，原发EGFR T790M的突变率波动于0.4%-66%^[3,4]^；在未经治疗的EGFR突变阳性的NSCLC中，原发T790M的突变率波动于1.07%-77.9%^[5,6]^。

但是，对于原发EGFR T790M突变的NSCLC的一线治疗尚缺乏大型临床研究及指南推荐。在临床实践中，由于三代EGFR-TKIs可同时靶向EGFR敏感突变及T790M突变^[7]^，一线治疗倾向首选三代EGFR-TKIs，但疗效尚待验证。本文中我们报道了3例晚期NSCLC伴EGFR敏感突变合并原发T790M突变患者接受阿美替尼联合贝伐珠单抗的治疗经验，同时总结了奥希替尼治疗NSCLC伴原发EGFR T790M突变的研究进展。

## 1 病例资料

病例1，女，45岁。2020年9月因“呼吸困难，右侧胸背疼痛”就诊，完善检查考虑右肺恶性肿瘤伴纵隔淋巴结和多发脑转移。患者因意识障碍无法行有创组织活检，血液基因检测：EGFR p.L858R（5.09%）、EGFR p.T790M（6.15%）。2020年9月-2021年8月一线共予阿美替尼+贝伐珠单抗14个周期（图1）。用药1周后患者神志好转，谵妄消失，呼吸困难及癌痛明显缓解。卡氏评分（Karnofsky performance status, KPS）从60分提高至80分。2个周期后评估，肺内病灶及颅内病灶均评价疗效：部分缓解（partial response, PR），8个周期后达最佳疗效（↓60.9%）。14个周期后评价疗效：肺内疾病进展（progressive disease, PD）（↑32.5%），颅内维持PR，无进展生存期（progression-free survival, PFS）为11.2个月。2021年8月完善经皮肺穿刺活检，病理阴性。穿刺标本（暗红色血性液体）基因检测：EGFR p.L858R（35.84%）、EGFR p.T790M（39.18%）、EGFR p.L718Q（11.77%）。2021年9月-2021年12月予二线阿美替尼+阿法替尼，最佳疗效为肺内病灶稳定（stable disease, SD），颅内维持PR。2021年12月评价疗效：肺内（47.5%↑）及颅内（41.9%↑）病灶均PD（图1）。PFS_2_为4个月。阿美替尼用药期间未见明显不良反应。

**图1:**
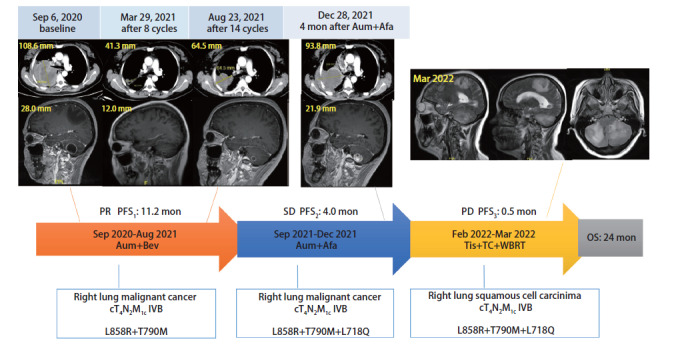
病例1的治疗过程及靶病灶的变化

2022年1月再次经皮肺穿刺活检，病理回报为鳞状细胞癌。三线予替雷利珠单抗+白蛋白紫杉醇+卡铂，同步颅脑放疗，计划10次（实际完成5次）。第1周期同步放化疗期间患者突发左侧肢体无力，头核磁示颅内病灶PD（图1）。患者家属放弃进一步治疗，自动出院，于2022年7月中旬去世，总生存期（overall survival, OS）约24个月。

病例2，女，58岁。既往乙肝、肝硬化病史。2021年4月因“左额顶部肿物”就诊，完善影像学检查，考虑右肺恶性肿瘤伴左侧腋窝、右肺门及纵隔内多发淋巴结转移，右侧胸膜、肝右叶后下段、骨、脑转移。2021年6月行“左额大脑病损切除术+颅骨缺损修补术”，病理回报：转移性腺癌，考虑来自肺。组织基因检测：EGFR 19del（21.82%）、T790M（21.57%）。2021年8月-2021年10月予阿美替尼联合贝伐珠单抗，最佳疗效胸部、颅内病灶PR，肝病灶SD（图2）。因肝硬化、脾静脉高压，出血风险高，2021年11月停用贝伐珠单抗，继续口服阿美替尼。2022年2月评价疗效：胸部、颅内病灶维持PR，肝右下极结节PD（图2），PFS_1_为7个月。进展后肝转移灶行射频消融治疗，并继续口服阿美替尼。2022年5月因个人原因更换为奥希替尼。末次影像学复查时间为2022年7月初，PFS_2_为5^+^个月。阿美替尼用药期间未见明显不良反应。

**图2:**
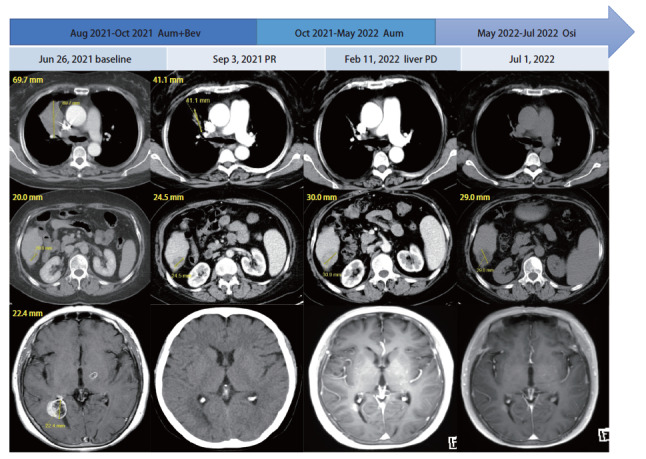
病例2的治疗过程及靶病灶的变化

病例3，女，50岁。2019年10月查体发现右肺上叶占位，行“右侧胸腔镜探查、右肺上叶楔形切除、术中冰冻、右肺上叶切除、纵隔淋巴结清扫术”，术后病理：（右肺上叶）浸润性腺癌，分期为pT_2a_N_0_M_0_ IB期，术后定期复查。2021年3月出现右侧胸痛，胸部计算机断层扫描（computed tomography, CT）示右残肺软组织肿块，新见两肺多发结节。血液基因检测：EGFR 19del（p.L747_P753delinsS, 0.57%）、T790M（1.34%）。于2021年4月-2022年4月行阿美替尼联合贝伐珠单抗治疗。2个周期后评价疗效：PR（图3）。2022年5月因个人原因更换为奥希替尼，并停用贝伐珠单抗。末次复查时间为2022年10月底，肺部病灶较前增大，但未见远处转移，综合评价疗效：缓慢进展。继续奥希替尼治疗，PFS为19^+^个月（图3）。阿美替尼治疗期间发生2度口腔炎，1度谷丙转氨酶（alanine transaminase, ALT）升高，对症处理后均好转。

**图3:**
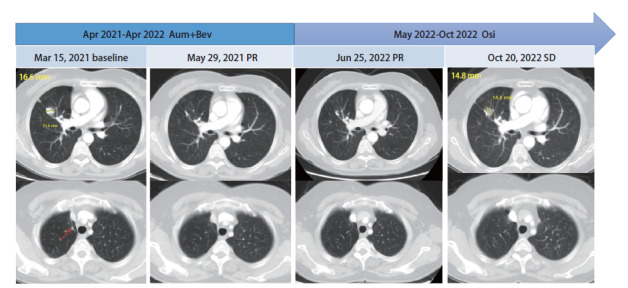
病例3的治疗过程及靶病灶的变化

## 2 讨论

目前三代EGFR-TKIs治疗NSCLC伴原发EGFR T790M突变的数据主要来自于奥希替尼的小样本回顾性研究（表1）。不同研究之间的数据波动较大，客观缓解率（objective response rate, ORR）波动于10.0%-72.2%，疾病控制率（disease control rate, DCR）波动于77.8%-100.0%，中位PFS波动于5.1个月-18.0个月，中位OS波动于22.4个月-27.7个月。在个案报道中，奥希替尼治疗NSCLC伴原发EGFR T790M突变也证实有不错的反应，但是疗效可能与伴随的EGFR突变类型有一定关系（表2）。

**表1:** 奥希替尼治疗原发T790M突变的文献总结

References	Type	Number of patients	ORR	DCR	mPFS (mon)	mOS (mon)
Zhang et al. 2018^[8]^	Retrospectively	15	NA	NA	18.0	25.1
Wang et al. 2019^[5]^	Retrospectively	18	72.2%	100%	17.0	29.9
Si et al. 2022^[9]^	Retrospectively	11	10.0%	NA	12.2	25.3
Zeng et al. 2022^[10]^	Retrospectively	9	55.6%	77.8%	NA	NR
Chang et al. 2022^[11]^	Retrospectively	5	60.0%	80.0%	5.1	22.4
Panda et al. 2022^[12]^	Retrospectively	14	64.3%	92.9%	14.1	27.7

ORR: objective response rate; DCR: disease control rate; NA: not available; NR: not reached

**表2:** 奥希替尼治疗原发T790M突变的个案报道总结

References	Age (yr)	Gender	Pathology	EGFR mutation	Stage	Metastasis	Response	PFS (mon)	OS (mon)	AE
Ancevski Hunter et al. 2018^[13]^	64	Female	AD	19del+T790M	IVB	NA	PR	8^+^	8^+^	-
Ancevski Hunter et al. 2018^[13]^	33	Female	AD	G719A+T790M	IVB	Liver	PR	5^+^	5^+^	-
Ancevski Hunter et al. 2018^[13]^	51	Male	AD	G719A+T790M	IVB	Brain	SD	15	15^+^	-
Ikari et al. 2019^[14]^	70	Male	AD	G719A+T790M	IVA	Pleural	PR	4^+^	4^+^	-
Häntschel et al. 2020^[15]^	79	Male	NSCLC	L858R+T790M	IVB	Adrenal gland, bone	PR	10	24^+^	ILD
Sumi et al. 2021^[16]^	76	Female	AD	L858R+T790M	IVB	Brain, adrenal gland, bone	PR	26^+^	26^+^	Rash
Sumi et al. 2021^[16]^	89	Male	AD	19del+T790M	IVB	Pleural, liver, bone	PR	24^+^	24^+^	-
Ito et al. 2022^[17]^	71	Female	AD	G719S+T790M	IVB	Brain	PR	5	8	-
Zeng et al. 2022^[10]^	71	Male	NSCLC	L861Q+T790M	IV	NA	PD	3.4	3.4^+^	-

EGFR: epidermal growth factor receptor; AD: adenocarcinoma; NSCLC: non-small cell lung cancer; ILD: interstitial lung disease.

相比奥希替尼，其他三代EGFR-TKIs对EGFR原发T790M突变的疗效缺乏报道。阿美替尼为我国新型不可逆三代EGFR-TKIs，在AENEAS研究^[18]^中阿美替尼对比吉非替尼一线治疗EGFR敏感突变局部晚期或转移性NSCLC可明显延长中位PFS（19.3个月 vs 9.9个月，HR=0.46，95%CI：0.36-0.60，P<0.0001）。WJOG9717L研究^[19]^中奥希替尼联合贝伐珠单抗一线治疗EGFR敏感突变的局晚或转移性非鳞癌NSCLC较奥希替尼单药并未改善PFS（22.1个月 vs 20.2个月，HR=0.862，95%CI：0.531-1.397，P=0.213）。但是，阿美替尼联合贝伐珠单抗一线治疗EGFR敏感突变的晚期NSCLC的有一定疗效。我们团队研究者发起的一项阿美替尼联合贝伐珠单抗一线治疗EGFR突变晚期NSCLC的单臂、单中心、II期临床研究，截止到2022年4月，共招募了19例患者，其中15例可评估疗效，总人群ORR为66.7%，DCR为93.3%。其中12例基线脑转移患者ORR为83.3%，8例脑转移可行颅内靶病灶评估，颅内ORR为87.5%，颅内DCR为100.0% ^[20]^。

本文中我们报道了3例晚期NSCLC伴EGFR敏感突变及原发T790M突变的治疗经验。3例患者在初始治疗后靶病灶评估最佳疗效均达PR，2例患者一线治疗后进展，PFS分别为11个月和7个月；1例患者治疗后持续反应，治疗时间已达19个月。2例患者基线伴有多发脑转移，颅内病灶最佳疗效均为PR，颅内PFS分别为14个月和未达到（16^+^个月）。

病例1是在病情危重且无法明确病理诊断的情况下依据血液检测结果一线予阿美替尼联合贝伐珠单抗治疗，用药1周后患者临床症状即得到明显改善，一线肺内PFS为11.2个月。该患者一线PFS较AENEAS研究的数据短，考虑主要有3个原因：一是该患者突变亚型为L858R，疗效差于EGFR 19del患者^[18]^；二是病理类型为鳞癌，疗效差于肺腺癌患者^[21]^；三是合并原发T790M突变可能会影响EGFR-TKIs的疗效（表1）。一线治疗后患者出现EGFR L718Q突变，EGFR L718Q/V突变约占奥希替尼耐药突变的7.3%-9.7%^[22]^。体外实验^[23]^表明对于L858R/L718V突变，阿法替尼的敏感性最佳，可能是因为阿法替尼结合方式受到L718V的阻碍最小。病案报道^[24]^也提示阿法替尼在体内对奥希替尼耐药后出现L718Q有一定反应。该患者二线探索性应用阿美替尼联合阿法替尼，最佳疗效为SD，PFS为4个月。这提示L718Q突变也是阿美替尼耐药机制之一，阿法替尼对L718Q突变的疗效仍待验证。

病例2一线予阿美替尼联合贝伐珠单抗治疗后肺内及颅内病灶均达到PR，尤其是颅内病灶接近CR状态，但是肝内病灶最佳疗效仅为SD，且在7个月后出现肝PD。患者为寡进展，依据美国国立综合癌症网络（National Comprehensive Cancer Network, NCCN）及中国抗癌协会临床肿瘤学协作专业委员会（Chinese Society of Clinical Oncology, CSCO）指南，寡进展病灶给予局部处理同时继续口服阿美替尼3个月，后因个人原因更换为奥希替尼，整体PFS达12^+^个月。

病例3为肺腺癌术后原位复发，血液检测为EGFR 19del伴原发T790M突变，一线予阿美替尼联合贝伐珠单抗治疗后达到PR，口服阿美替尼13个月后因个人原因更换为奥希替尼。2022年10月底复查肺部病灶较前增大，未见远处转移。考虑患者目前的疾病控制时间已超过6个月；仅局部病灶增大，考虑奥希替尼仍有临床获益；靶病灶增大有限；且患者无相关临床症状。符合EGFR-TKIs治疗后缓慢进展模式，予继续口服奥希替尼^[25]^。一线整体PFS为19^+^个月。

病例1及病例3均是依据血液循环肿瘤细胞DNA（circulating tumor DNA, ctDNA）基因检测给予EGFR-TKIs靶向治疗。BENEFIT研究^[26]^基于血液检测到IV期肺腺癌患者EGFR敏感突变给予吉非替尼一线治疗，ORR为72.1%（95%CI: 65.0%-78.5%），中位PFS为9.5个月（95%CI: 9.07-11.04）。若无法病理确诊肺恶性肿瘤，依据血液检测是否能指导临床治疗，Challenge研究^[27]^给出了答案，该研究旨在评估在无法获得病理诊断且血液检测到EGFR敏感突变的临床诊断晚期肺癌患者中接受一线埃克替尼治疗的疗效。结果显示，血液EGFR突变指导的一线埃克替尼的ORR为52.6%，DCR为84.5%，中位PFS为10.3个月，中位OS为23.2个月。因此，在无法获取病理诊断时，液体活检的分子诊断也可以作为临床决策制定和精准治疗选择的依据，使得更多患者获得精准治疗的机会。

在安全性方面，病例1、2未见明显不良反应，病例3在治疗期间发生2度口腔炎，1度ALT升高，对症处理后均好转。我们团队研究者发起的阿美替尼联合贝伐珠单抗一线治疗EGFR突变晚期NSCLC的单臂、单中心、II期临床研究中3级不良反应的发生率为15.8%，主要为无症状肌酸激酶升高（10.5%），1例患者因胸痛停用贝伐珠单抗^[20]^。可见，阿美替尼联合贝伐珠单抗不良反应可控，总体耐受性良好。

## 3 总结

目前关于原发EGFR T790M突变的研究数据多来源于小样本回顾性研究，多数未考虑共突变/复合突变，且由于检测方法不一导致原发T790M的检出率相差较大，可能造成研究结果的偏倚。因此，原发EGFR T790M突变的治疗需要更多循证医学证据的支持。本研究的3个病例显示，阿美替尼联合贝伐珠单抗治疗晚期NSCLC伴原发EGFR T790M突变具有较高的ORR及对脑转移的控制能力，可作为一线初始治疗的选择之一。
